# Integrating robotic surgery training and the educational value of body donation: insights from an Italian pilot project on perceived training value

**DOI:** 10.3389/fmed.2026.1907504

**Published:** 2026-08-26

**Authors:** Irene Neri, Laura Forni, Simone Lodi, Clarissa A. E. Gelmi, Stefano Leto, Elisa Boschetti, Matteo Serenari, Lorenzo Bianchi, Paolo Bernante, Matteo Rottoli, Riccardo Schiavina, Matteo Cescon, Elio Jovine, Lucia Manzoli, Stefano Ratti

**Affiliations:** 1 Cellular Signalling Laboratory, Anatomy Center, Department of Biomedical and Neuromotor Sciences (DIBINEM), University of Bologna, Bologna, Italy; 2 Department of Medical and Surgical Sciences (DIMEC), University of Bologna, Bologna, Italy; 3 Hepatobiliary Surgery and Transplant Unit, IRCCS Azienda Ospedaliero Universitaria di Bologna, Bologna, Italy; 4 Urology Unit, IRCCS Azienda Ospedaliero Universitaria di Bologna, Bologna, Italy; 5 Center for Metabolic and Obesity Surgery, IRCCS Azienda Ospedaliera Universitaria di Bologna, Bologna, Italy; 6 Surgery of the Alimentary Tract, IRCCS Azienda Ospedaliero-Universitaria di Bologna, Bologna, Italy

**Keywords:** da Vinci Xi platform, full body donation, human anatomy, medical education, surgical simulation

## Abstract

**Background:**

Robotic-assisted surgery requires training that integrates technical skills, visuospatial coordination, platform management, and anatomical understanding. Although synthetic and virtual-reality simulators are widely used, high-fidelity training on bodies donated to science remains comparatively underreported. This pilot study therefore aimed to evaluate participants’ perceived value of a robotic surgery training experience with the da Vinci Xi platform in a body donation setting, and to examine whether this perceived value was associated with participants’ views on the educational role of body donation.

**Methods:**

This pilot post-course mixed-methods evaluation involved 41 residents and surgeons in general surgery and urology. The program combined dry-lab and wet-lab activities using the da Vinci Xi platform and three body donors. Participants completed a study-specific questionnaire including seven Likert-type items and two open-ended questions. Quantitative data were analyzed descriptively, through Spearman correlations and exploratory subgroup comparisons; qualitative responses underwent thematic analysis.

**Results:**

Course evaluations ratings were highly positive, with mean scores ranging from 4.59 to 4.88 on a 5-point scale. The mean Training Value Index was 4.70, and the five course evaluation items showed good internal consistency. All participants expressed high willingness to attend a similar course again, and 97.6% would recommend it to colleagues. Agreement that body donation represents a gold standard for medical and surgical education and training, and its perceived ethical importance, received high ratings from 95.1 and 97.6% of participants, respectively. Both were significantly associated with the Training Value Index. Qualitative feedback highlighted hands-on robotic practice, anatomical fidelity, operative-like workflow, and requests for longer or repeated training opportunities.

**Conclusion:**

Participants perceived robotic surgery training in a body donation setting as highly valuable, and this perception was closely linked to their broader recognition of the educational value of body donation. These preliminary findings suggest that combining technical training, anatomical complexity, team-based workflow, and ethical awareness may represent a promising complement to dry-lab and virtual-reality simulation, warranting further investigation to inform the development of more integrated robotic surgery curricula.

## Introduction

Technological innovation has profoundly reshaped contemporary surgical practice, increasing procedural precision while also making surgical training more complex. This is particularly evident in robotic-assisted surgery, where trainees must acquire not only anatomical and procedural knowledge, but also console-based skills, visuospatial coordination, instrument control, and familiarity with technologically mediated interaction with the operative field (1, 2). For these reasons, surgical education can no longer rely exclusively on traditional apprenticeship models, especially in a context where structured training and patient safety are increasingly central (3). More broadly, simulation-based education has become a key component of modern medical training because it allows learners to practice in controlled environments without exposing patients to avoidable risk, while providing opportunities for deliberate practice with structured feedback (4, 5).

Despite its recognized value, simulation-based training remains limited by the characteristics of the models available. Synthetic simulators allow repetition and standardization, but often provide limited anatomical fidelity (6). Animal models may offer procedural realism, yet they are constrained by ethical concerns, high costs, and anatomical differences that reduce transferability to human surgery (6, 7). Human body donor-based training models, by contrast, remain uniquely valuable because they provide direct access to authentic anatomical relationships, tissue planes, and procedure-relevant spatial complexity, making them especially important in advanced surgical education (6–8).

Body donation to science constitutes a foundational scientific, educational, and ethical resource, enabling anatomical teaching, procedural practice, translational research, and innovative training environments (9, 10). Body donors are recognized as “silent teachers,” whose contribution deserves respect and acknowledgment (11, 12). Importantly, recent evidence suggests that locally sourced donations also carry a lower environmental footprint than internationally transported specimens, reinforcing the value of local donation programs (13).

This is particularly relevant for centers with a well-established infrastructure for receiving and managing bodies donated to science. An example is the Anatomy Center at the University of Bologna, where a Program for post-mortem body donation was launched as early as 2013, ahead of Law No. 10/2020, the first Italian legislation governing the post-mortem donation of bodies and tissues for the purposes of study, training and scientific research on a national scale. In 2021, the Center was recognised by the Italian Ministry of Health as a National Reference Center (14, 15). Building on this foundation, the Center has progressively supported advanced forms of surgical simulation, including a SimLife®-based reperfusion and re-ventilation experience demonstrating that technology can amplify, rather than replace, the educational value of body donation (16).

Among the fields that may benefit most from this approach is robotic-assisted surgery using the da Vinci surgical system. The da Vinci system allows surgeons to control instruments from a console, translates the surgeon’s hand movements in real time, uses wristed instruments with a greater range of motion than the human hand, and provides highly magnified three-dimensional high-definition views of the surgical field. These features make training dependent not only on technical repetition, but also on adaptation to a specific operative interface and on the integration of anatomical knowledge with console-based performance (1, 2). The learning curve for robotic surgery demonstrates continuous improvement throughout the first 100 surgeries, with efficiency in surgical times typically attained after 20–30 cases, underscoring the importance of structured training environments (17).

Given these training requirements, it is especially relevant to consider how training with the da Vinci system has been implemented within high-fidelity anatomical settings. While the educational value of training on donated human bodies is increasingly recognized in surgical education (8), only limited evidence has specifically addressed its integration with robotic surgery training using the da Vinci system. In this regard, an isolated single-session cadaver-based experience for urology residents in the USA explicitly referred to da Vinci-related skill domains, suggesting that donated human bodies may also support this form of technologically advanced surgical education (18). At the same time, a recent systematic review of 209 studies confirmed that the da Vinci Surgical System and the da Vinci Skills Simulator are among the predominant tools used in robotic training, particularly in dry lab and virtual reality settings, respectively, (1).

Against this background, the present pilot study aimed to explore participants’ evaluation of a robotic surgery training program using the da Vinci Xi platform, conducted at the Anatomy Center of the University of Bologna, a nationally recognized reference center for body donation that provided the infrastructure for a high-fidelity simulation experience. In particular, the study focused on overall course appraisal, perceived educational value, and qualitative feedback from attendees. In doing so, it also aimed to extend a line of inquiry previously initiated at the same institution, namely that technological innovation in surgical simulation and body donation are not competing paradigms but mutually reinforcing ones, to the specific and underexplored domain of robotic surgery training using the da Vinci system.

## Materials and methods

### Simulation setting and participants

The Anatomy Center of the University of Bologna hosts two fully equipped dissection rooms: the main dissection room, equipped with four workstations and an audio-video-endoscopic system for recording and streaming activities, and a high-technology anatomical room with a modular system for two surgical workstations. The present initiative was specifically organized around the da Vinci Xi platform and took place in the main dissection room. The course was delivered over a three-week period and involved three body donors provided through the national body donation program, in accordance with applicable legal and ethical requirements.

Upon arrival at the Anatomy Center, all body donors underwent computed tomography (CT) scanning using the Philips Incisive CT 128 system available at the Center. This multislice CT scanner allows helical image acquisition and three-dimensional reconstruction of anatomical regions, including organs, soft tissues, and skeletal structures. Body donors arrived at the Center fresh and were subsequently frozen at −20 °C following CT scanning. Approximately one week before each simulation week, donors were thawed at room temperature. As each donor was used across three consecutive days (Wednesday to Friday), donors were stored at 4 °C overnight between sessions to preserve tissue quality throughout the week of use.

Before the simulation sessions, participants attended a dedicated briefing in which the morphological and radiological characteristics of each body donor were discussed. This briefing was intended to support anatomical orientation, procedural planning, port-placement reasoning, and safe interaction with the donated bodies during the simulation activities. Moreover, prior to the practical activities, all participants attended an introductory session on the regulatory framework (Italian Law 10/2020) and the ethical significance of body donation, to ensure full awareness of the voluntary nature of donors’ contribution.

**Figure 1:**
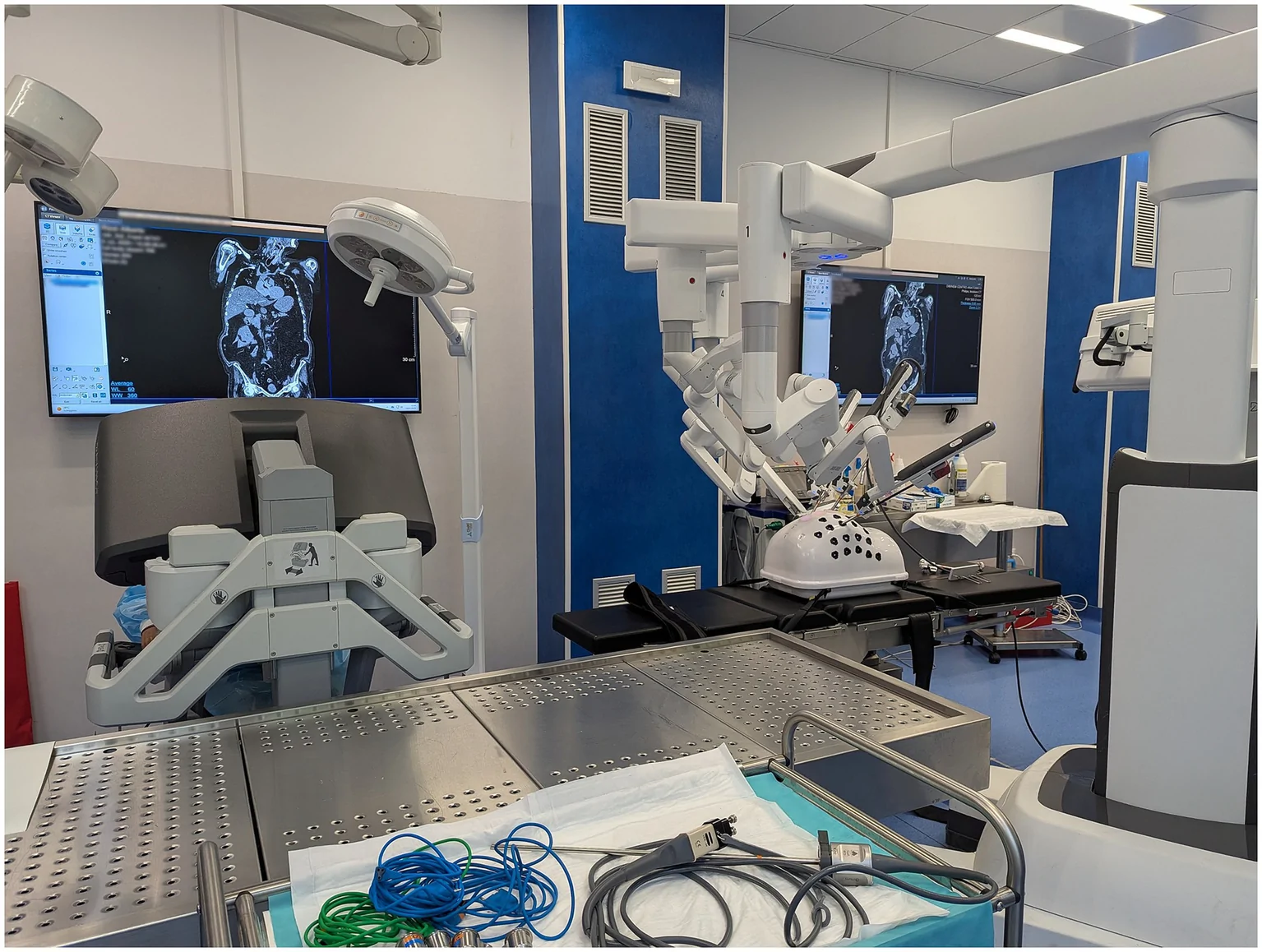
Dry-lab training set-up in the Mazzotti Room of the Anatomy Center of the University of Bologna. The da Vinci Xi patient cart is shown docked onto a pelvic trainer, with the room monitors displaying pelvic CT imaging in the background. The surgical console is visible on the left.

**Figure 2:**
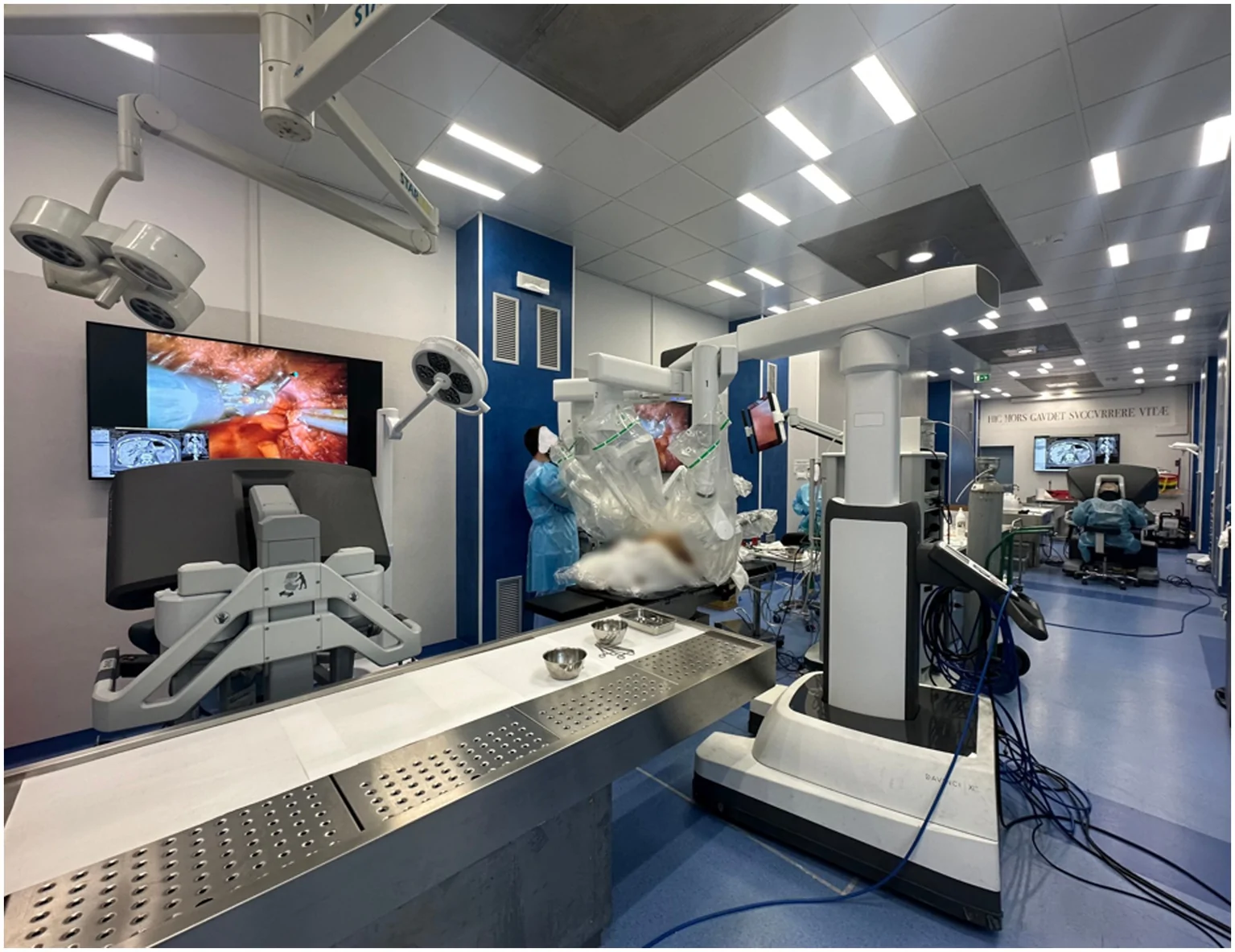
Wet-lab training set-up in the Mazzotti Room of the Anatomy Center of the University of Bologna. The da Vinci Xi surgical console (Station 1) is visible in the foreground on the left, while a participant managing table assistance (Station 2) is visible in the background alongside the docked patient cart. The room monitors display the live endoscopic view of the operative field. In the far background, the simulation console used for independent practice is visible.

The course was conducted in English, and the training pathway combined dry-lab and wet-lab activities. The dry-lab component consisted of simulator- and bench-based practice without human tissue, aimed at building familiarity with the robotic platform before progressing to wet-lab activities involving body donors. It was conducted using both a digital simulator, running Intuitive Surgical’s proprietary simulation software (da Vinci Skills Simulator), and a physical pelvic trainer used without dedicated software, and was structured around basic robotic tasks such as docking, port placement, instrument management, collision control, initial set-up, and simulator-based exercises, including EndoWrist manipulation and camera control (Figure 1). The wet-lab component was carried out on body donors and involved complex surgical procedures relevant to each specialty, including nephrectomy and prostatectomy for urology-focused sessions, and esophagectomy, partial bowel resection and hepatic resection for general-surgery-focused sessions. As an illustrative example, the robotic-assisted partial nephrectomy simulation involved renal exposure and mobilization, hilar dissection, vascular control, and renorrhaphy, with participants at Station 1 executing the console-based dissection and suturing steps and participants at Station 2 providing table assistance, including instrument exchange and retraction. Sessions were organized into urology-focused and general-surgery-focused sessions, with rotation through two operative stations: Station 1 (surgical console) and Station 2 (table assistance, including aspiration management and robotic arm management). The operating-room set-up involved the patient cart, vision cart, and surgeon console, and participants were trained not only in console-based execution but also in intraoperative robotic management and team positioning around the platform. In addition, a simulation console with preloaded simulation software was available in the room to allow participants to practice robotic arm control independently of the surgical console (Figure 2).

The course involved 41 participants: 35 residents (19 in general surgery, 16 in urology) and 6 surgeons (4 in general surgery, 2 in urology), who also served as supervisors for the wet-lab sessions. All resident participants had prior dry-lab experience with the da Vinci simulator and had observed live robotic-assisted procedures, but had not previously performed robotic surgery independently. Supervising surgeons held senior academic and clinical positions in general surgery and urology, with established experience in robotic-assisted surgery, and had a pre-existing training relationship with the resident participants in their usual clinical and academic setting. Sessions were organized on a weekly basis as summarized in Table 1. Dry-lab sessions (Monday and Tuesday) accommodated 6 participants per group, with sessions dedicated exclusively to either general surgery or urology residents. Wet-lab sessions ran from Wednesday to Friday, with urology-focused groups on Wednesday and general-surgery-focused groups on Thursday and Friday, accommodating 2 participants per group. Dry-lab sessions were supervised by an AB Medica technical support specialist, responsible for instructing participants in the manual operation of the robotic platform, while one supervising surgeon, drawn from among the 6 attending surgeons enrolled in the course, was present for each wet-lab group at all times. Each session lasted 3 h, with participants rotating between Station 1 and Station 2 every 1.5 h. In week 3, the final wet-lab group (Friday afternoon) consisted of 1 resident paired with 1 senior surgeon, while all other wet-lab groups throughout the course followed the standard format of 2 residents under the supervision of 1 surgeon. From an individual participant’s perspective, each trainee attended a single 3 h dry-lab session on Monday or Tuesday, followed by a single 3 h wet-lab session later in the week (Wednesday for urology, Thursday or Friday for general surgery), during which they rotated between Station 1 and Station 2 at the 1.5 h mark. Each participant thus received a total of 6 h of hands-on training over the course of a single week.

**Table 1:** Example weekly training schedule.

Session	Monday(dry lab)	Tuesday(dry lab)	Wednesday(wet lab URO)	Thursday(wet lab GS)	Friday(wet lab GS)
Morning 09:00–12:00	6 residents GS or URO 1 supervisor	6 residents GS or URO 1 supervisor	2 residents URO 1 supervisor	2 residents GS 1 supervisor	2 residents GS 1 supervisor
Afternoon 13:00–16:00	6 residents GS or URO 1 supervisor	6 residents GS or URO 1 supervisor	2 residents URO 1 supervisor	2 residents GS 1 supervisor	2 residents GS 1 supervisor

The course spanned three weeks in total; the schedule shown represents one representative week, with the same pattern repeated using a different body donor each week. GS = general surgery; URO = urology.

### Study design and questionnaire development

This study was designed as a pilot post-course evaluation study based on a cross-sectional questionnaire administered after completion of the training experience. A mixed-methods descriptive approach was adopted, combining structured quantitative items with open-ended questions in order to capture both measurable course evaluation and participants’ subjective expectations and feedback.

The questionnaire was specifically developed for the present study to explore whether a robotic simulation experience conducted with the da Vinci Xi platform in a body donation setting would be perceived as educationally valuable, capable of meeting participants’ expectations, and relevant to the development of robotic surgical skills. It also aimed to investigate whether participants valued the course in relation to the realism of the simulation environment and the broader educational relevance of body donation-based training.

In addition to demographic variables (age, gender, and nationality), the questionnaire collected training-related information, including professional status, surgical specialty, and previous experience with activities involving bodies donated to science. The study-specific questionnaire then comprised nine items. Q1 was an open-ended question investigating participants’ expectations regarding the course. Q2–Q8 comprised seven Likert-type items, grouped into two sections. The first section (Q2–Q6) assessed participants’ evaluation of the course itself: overall course rating, whether the course met expectations, perceived usefulness for skills development, willingness to attend a similar course again, and likelihood of recommending this type of course to a colleague. The second section (Q7–Q8) assessed participants’ perception of body donation as an educational resource: agreement with the statement “Body donation represents a gold standard for medical and surgical education and training,” and the perceived ethical importance of body donation for medical and surgical education and training. All Likert-type items were rated on a 5-point scale. Q9 was an open-ended question designed to collect post-course comments and suggestions. The full set of questionnaire items is provided in the Supplementary material. *Verbatim* participant responses to the two open-ended items (Q1 and Q9) are also provided in the Supplementary material.

### Coding of variables and statistical analysis

SPSS statistical package, version 25.0 (IBM Corp., Armonk, NY, USA) was used to statistically analyze the collected data. Demographic and training-related variables were coded in a structured form to allow statistical analysis.

Descriptive statistics were calculated for all variables. Demographic and training-related variables were summarized as frequencies and percentages, whereas the seven Likert-type items (Q2–Q8) were described using mean, standard deviation, median, and interquartile range. Internal consistency of the five course evaluation items (Q2–Q6) was assessed using Cronbach’s alpha, and a composite Training Value Index (TVI), summarizing participants’ overall evaluation of the course across its core dimensions (overall rating, expectations met, perceived usefulness, willingness to repeat, and likelihood to recommend), was then calculated as the mean of these five items.

Associations among the five course evaluation items, the TVI, and age were explored using Spearman’s rank correlation coefficient (*ρ*). Spearman’s rank correlation coefficient was also used to explore associations between the two body donation perception items (Q7 and Q8) and each of the five course evaluation items (Q2-Q6), as well as the correlation between Q7 and Q8 themselves. Exploratory comparisons of the TVI and of the body donation perception items (Q7 and Q8) across subgroups defined by gender, nationality, professional status, specialization, and previous experience with activities involving bodies donated to science were performed using the Mann–Whitney U test. For descriptive purposes, responses to the seven Likert-type items were also dichotomized into high ratings (scores 4–5) versus lower ratings (scores 1–3). A *p*-value < 0.05 was considered statistically significant.

The questionnaire coding scheme and the complete SPSS outputs are provided in the Supplementary material.

### Qualitative analysis of open-ended responses

Open-ended responses were analyzed using thematic analysis following the framework described by Kiger and Varpio (19). Two researchers independently coded participants’ responses; codes were then compared, and discrepancies were resolved through discussion and consensus, without a formal quantitative measure of inter-coder agreement. Themes were derived directly from the coded responses, identifying common topics, ideas, and patterns of meaning, which were subsequently organized into macro-themes for a comprehensive understanding of the data.

## Results

### Participant characteristics

A total of 41 participants were included in the analysis. Of these, 23 (56.1%) were male and 18 (43.9%) were female. Most participants were Italian (39, 95.1%), while 2 (4.9%) were classified as non-Italian (1 Moldavian and 1 Jordan). Regarding professional status, 35 (85.4%) were residents and 6 (14.6%) were surgeons. In terms of specialization, 23 (56.1%) were general surgeons and 18 (43.9%) were urologists. Previous experience with courses involving bodies donated to science was reported by 32 participants (78.0%), whereas 9 (22.0%) reported no previous experience. Table 2 summarizes participants’ characteristics.

**Table 2:** Participants’ demographics and training information.

Demographics	Number of participants
Gender	
Female	18
Male	23
Age	
25–30	14
31–35	21
36–40	2
>40	4
Nationality	
Italian	39
Moldavian	1
Jordan	1
Occupation	
Resident	35
Surgeon	6
Specialization	
General surgeon	23
Urologist	18
Previous experience with donated bodies	
Yes	32
No	9
	Total = 41

### Descriptive evaluation of the course

Overall, participants reported highly positive responses across the five course evaluation items and the two body donation perception items, all rated on a 5-point scale. Course evaluation and body donation perception items were rated consistently highly, with mean scores ranging from 4.54 to 4.88 and median values of 5.00 for all items, indicating a pronounced ceiling effect. Full descriptive statistics (mean, SD, median, quartiles, IQR) for each item (Q2–Q8) are reported in Table 3.

**Table 3:** Likert questions descriptive statistics.

Items	Mean	SD	Median	25%	75%	IQR
Q2	4.59	0.81	5.00	4.00	5.00	1.00
Q3	4.59	0.95	5.00	5.00	5.00	0.00
Q4	4.61	0.77	5.00	4.50	5.00	0.50
Q5	4.88	0.33	5.00	5.00	5.00	0.00
Q6	4.85	0.42	5.00	5.00	5.00	0.00
Q7	4.54	0.60	5.00	4.00	5.00	1.00
Q8	4.73	0.50	5.00	4.50	5.00	0.50

**Figure 3:**
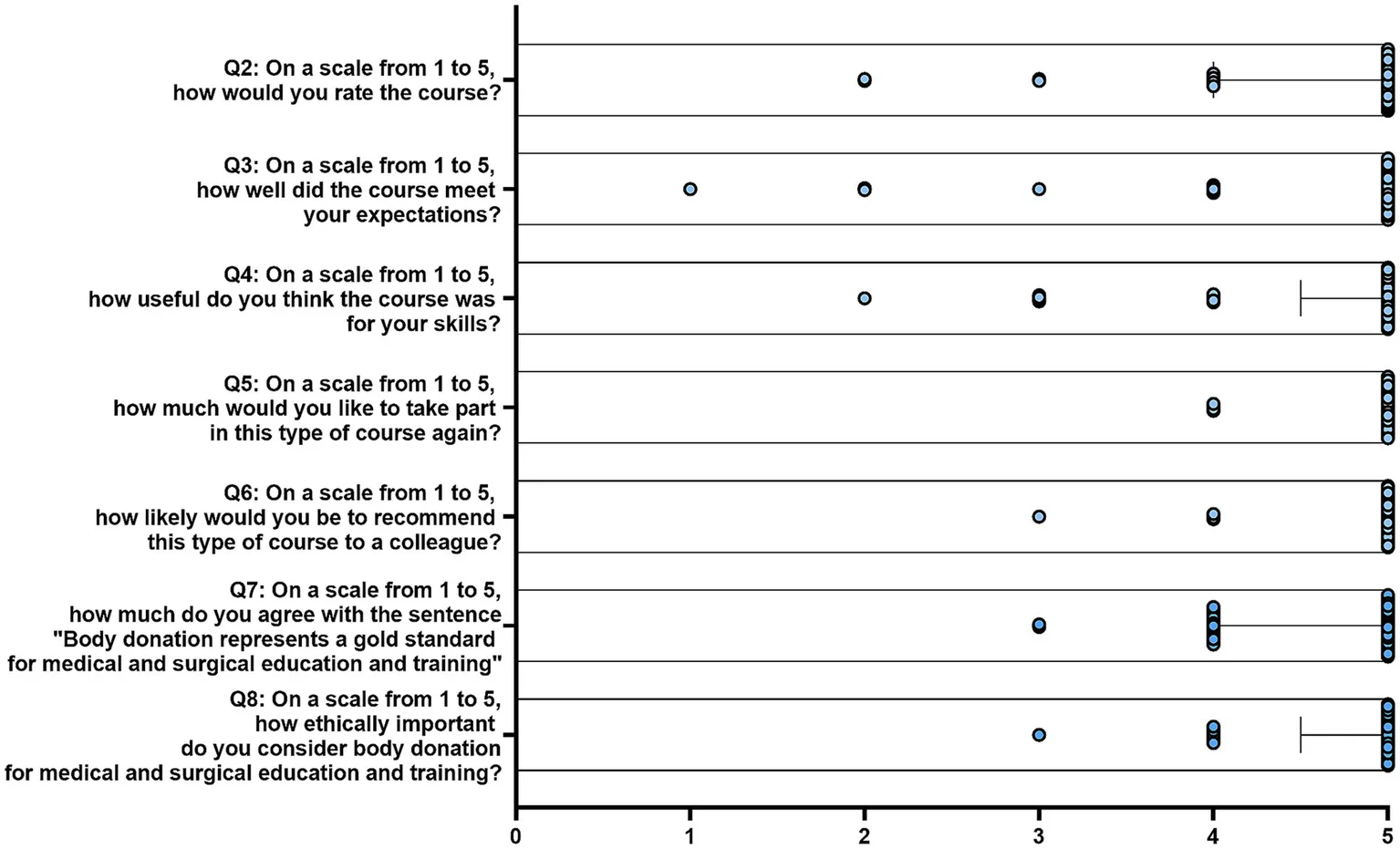
Distribution of responses across the seven Likert-type items. Dots represent individual responses; central bars indicate the median, and horizontal error bars indicate the interquartile range (IQR). Where not visible, the IQR was equal to 0.

As shown in Figure 3, responses were strongly skewed toward the most positive categories, with very limited dispersion across items.

Frequency distributions confirmed this highly positive pattern. For Q2, 90.2% of participants gave a high rating (4 or 5). Likewise, for Q3, 90.2% gave a high rating. For Q4, 87.8% gave a high rating. For Q5, all participants (100%) gave a high rating, while 97.6% did so for Q6. For Q7, 95.1% agreed or strongly agreed that body donation represents a gold standard for medical and surgical education and training. Finally, for Q8, 97.6% rated the ethical importance of body donation for medical and surgical education and training as very or extremely high.

### Internal consistency of the evaluation items

The five evaluation items showed good internal consistency, with a Cronbach’s *α* of 0.863 (standardized α = 0.876). This supported the use of a composite score summarizing participants’ overall evaluation of the course.

### Training value index

The TVI had a mean value of 4.70 (median = 5.00; IQR = 0.40).

### Correlation analyses

Spearman correlation analyses showed that the five evaluation items were positively associated with one another. In particular, Q2 strongly correlated with Q3 (*ρ* = 0.902, *p* < 0.001) and was also positively associated with Q4 (ρ = 0.396, *p* = 0.010), Q5 (ρ = 0.580, *p* < 0.001), and Q6 (ρ = 0.653, *p* < 0.001).

The composite TVI was strongly associated with all individual evaluation items: Q2 (ρ = 0.861, *p* < 0.001), Q3 (ρ = 0.812, *p* < 0.001), Q4 (*ρ* = 0.728, *p* < 0.001), Q5 (*ρ* = 0.560, *p* < 0.001), and Q6 (ρ = 0.625, *p* < 0.001).

By contrast, age was not significantly correlated with any of the evaluation items or with the TVI (all *p* > 0.05).

The two body donation perception items were also positively associated with participants’ evaluation of the course. Agreement with the statement that body donation represents a gold standard for medical and surgical education and training (Q7) was significantly correlated with all five course evaluation items, with Spearman’s *ρ* ranging from 0.460 to 0.566, and with the TVI (ρ = 0.636, *p* < 0.001). The perceived ethical importance of body donation for medical and surgical education and training (Q8) was significantly correlated with Q2 (ρ = 0.459, *p* = 0.003), Q3 (ρ = 0.426, *p* = 0.006), Q5 (ρ = 0.337, *p* = 0.031), Q6 (ρ = 0.509, *p* < 0.001), and the TVI (ρ = 0.462, *p* = 0.002), while its association with Q4 did not reach statistical significance (ρ = 0.295, *p* = 0.061). Q7 and Q8 were significantly correlated with each other (ρ = 0.559, *p* < 0.001).

Correlations among the seven Likert-type items, the composite Training Value Index, and age are reported in Table 4.

**Table 4:** Spearman’s correlation matrix.

Variable	Q2	Q3	Q4	Q5	Q6	Q7	Q8	TVI	Age
Q2	—								
Q3	0.902**	—							
Q4	0.396*	0.472**	—						
Q5	0.580**	0.501**	0.339*	—					
Q6	0.653**	0.728**	0.532**	0.527**	—				
Q7	0.551**	0.566**	0.472**	0.523**	0.460**	—			
Q8	0.459**	0.426**	0.295	0.337*	0.509**	0.559**	—		
TVI	0.861**	0.812**	0.728**	0.560**	0.625**	0.636**	0.462**	—	
Age	−0.059	−0.174	−0.165	0.098	−0.220	0.230	0.077	−0.113	—

Spearman’s ρ coefficients are reported. *p < 0.05, **p < 0.01.

### Exploratory subgroup analyses

Exploratory Mann–Whitney U tests were conducted to assess whether the TVI and the two body donation perception items differed according to participant characteristics.

For the TVI, a significant difference emerged by gender: male participants showed higher ranks than female participants (U = 115.50, Z = −2.738, *p* = 0.006). A significant difference also emerged by specialization, with participants in urology scoring higher than those in general surgery (U = 129.00, Z = −2.261, *p* = 0.024). No significant differences were found according to nationality (U = 23.00, Z = −1.103, *p* = 0.270), profession (U = 80.00, Z = −1.050, *p* = 0.294), or previous participation in courses involving bodies donated to science (U = 133.00, Z = −0.395, *p* = 0.693).

For the item assessing agreement with the statement that body donation represents a gold standard for medical and surgical education and training (Q7), a significant difference emerged by gender, with male participants showing higher ranks than female participants (U = 128.50, Z = −2.380, *p* = 0.017). No significant differences were found for Q7 according to nationality (U = 22.00, Z = −1.187, *p* = 0.235), profession (U = 73.50, Z = −1.341, *p* = 0.180), specialization (U = 203.50, Z = −0.015, *p* = 0.988), or previous experience with courses involving bodies donated to science (U = 134.00, Z = −0.364, *p* = 0.716).

For the item assessing the perceived ethical importance of body donation for medical and surgical education and training (Q8), no significant subgroup differences were observed according to gender (U = 192.00, Z = −0.528, *p* = 0.598), nationality (U = 29.00, Z = −0.811, *p* = 0.418), profession (U = 75.00, Z = −1.482, *p* = 0.138), specialization (U = 199.00, Z = −0.177, *p* = 0.859), or previous experience with courses involving bodies donated to science (U = 139.00, Z = −0.211, *p* = 0.833).

### Qualitative feedback from open-ended questions

Of the 41 participants, 25 (61.0%) responded to Q1 and 18 (43.9%) responded to Q9. Qualitative feedback from the open-ended questions was consistent with the quantitative evaluation. In Q1, participants’ expectations were mainly centered on two aspects: improvement of robotic technical skills and the possibility of experiencing a high-fidelity simulation environment close to the reality of the operating room. Notably, several participants framed this second expectation explicitly around the human dimension of the experience, describing their anticipation of working on human tissue (e.g., *“[I am expecting to have the] opportunity to practically apply the use of the robot on human tissue”*), of a simulation *“as close to reality as possible,”* or of their *“first experience at the robotic console on a human.”* In Q9, comments were largely positive and emphasized the educational value of the initiative, while the most recurrent suggestions concerned the need for more time for hands-on practice and the desirability of continuing or expanding similar training opportunities. The main themes emerging from Q1 and Q9 are summarized in Table 5.

**Table 5:** Main themes emerging from open-ended responses.

Open-ended question	Theme	Description	Illustrative quote
Q1	Improvement of professional skills	Desire to enhance technical competence and familiarity with robotic procedures	*“Deepen the use of the robot in general surgery and improve practical skills”*
Q1	High-fidelity simulation experience	Expectation of a realistic training experience based on human tissue, in an operative-like setting that only body donation can provide	*“Have a surgical experience as close to reality as possible”*
Q9	More time for hands-on training	Requests for longer practical sessions	*“I would increase the duration of the course”*
Q9	Repetition/continuity	Desire for additional editions or repeated opportunities	*“It would be fantastic to maintain this course also for senior residents in the coming years”*

## Discussion

Robotic surgery training requires educational environments that go beyond console repetition, integrating anatomical realism, operative workflow, and team-based practice. The present pilot study evaluated a robotic surgery training program using the da Vinci Xi platform and embedded within a formal body donation setting, addressing a gap in the literature on cadaveric simulation for robotic surgical education (8). Overall, participants reported a highly positive perception of the course. All seven Likert-type items showed high scores. The composite TVI was close to the maximum value, and the five course evaluation items showed good internal consistency, suggesting that the course was perceived as a coherent and valuable educational experience rather than as a set of isolated activities.

Among the course evaluation items (Q2-Q6), willingness to attend a similar course again and likelihood of recommending it to colleagues received the highest scores, indicating that participants perceived the format as credible and valuable. While the present course targeted residents and surgeons, adapted versions of body donation-based robotic simulation could plausibly be explored for undergraduate medical students, for whom early exposure to robotic platforms has been associated with greater comfort with the technology and increased interest in surgical careers (20–22). This remains speculative, however, as our data pertain exclusively to postgraduate trainees.

The open-ended responses help explain the perceived value of the course. Before the course, participants mainly expected to improve robotic technical skills and experience a simulation close to operating-room reality, often framing this explicitly around the human dimension, such as working on human tissue or having their “*first experience at the robotic console on a human*”. This points to what body donation uniquely provides: an anatomical realism that synthetic or virtual models cannot replicate, and which is central to the broader challenge of integrating console-based gestures, visuospatial coordination, and team interaction within a coherent operative workflow (23, 24). In this sense, the perceived value of the course was not an abstract appraisal of educational quality, but was grounded in specific, tangible elements of the experience, direct hands-on exposure to human tissue at the console and as table assistant, and the resulting sense of operative-like realism that participants explicitly contrasted with simulation on synthetic or virtual models.

Quantitative data corroborated this interpretation. Agreement that body donation represents a gold standard for surgical education (Q7) and its perceived ethical importance (Q8) were both rated highly, by 95.1 and 97.6% of participants respectively, and both items were significantly correlated with the TVI and with most individual evaluation items. Participants who more strongly endorsed body donation as educationally and ethically valuable also rated the course itself more highly, suggesting that participants’ evaluation of the course was positively associated with their recognition of the educational and ethical value of the body donation setting.

Post-course comments largely emphasized the educational value of the initiative, with the most recurrent suggestions concerning more hands-on time and the desirability of repeating or expanding the course. Rather than indicating a weakness, this likely reflects participants’ recognition of the specific value of direct engagement with the full operative configuration, including docking, instrument management, table assistance, and the anatomical conditions provided by body donation. Taken together, these findings suggest the course was valuable for two connected reasons: it offered a safe, structured environment for approaching robotic skills, while placing them within a high-fidelity anatomical context that dry-lab and virtual-reality modalities, despite their established value for repetition and standardization, cannot fully reproduce in terms of anatomical variability, spatial depth, and tissue-level realism (1, 25, 26).

Exploratory subgroup analyses offered further insight. No significant differences emerged according to previous experience with donated bodies, for either the TVI or the body donation perception items, suggesting that the course’s value was not restricted to those already familiar with donor-based training, while the robotic component appears to have added a distinctive layer even for experienced participants. Participants in urology reported higher TVI scores than those in general surgery. This difference should be regarded as exploratory and hypothesis-generating only, given the small sample size, multiple testing, and unmeasured confounders such as previous robotic experience, year of training, actual console time, and prior exposure to the da Vinci platform. One plausible contributing factor is the stronger historical integration of robotic platforms within urological practice (27, 28), though the broader and more heterogeneous training priorities of general surgery may also play a role (29, 30). The gender difference, with male participants reporting higher TVI and Q7 ranks than female participants, should instead be regarded as hypothesis-generating only. The study was not designed to investigate gender-related differences, and future studies should explore whether factors such as previous robotic exposure, self-efficacy, or differential access to operative opportunities influence perceived training value. Nationality was also included as a subgroup variable to explore whether it was associated with course evaluation or body donation perception; no significant differences emerged, but with only 2 non-Italian participants out of 41, this comparison carries limited statistical power and should likewise be regarded as exploratory only.

The main contribution of this study lies in describing and evaluating a robotic surgery training experience using the da Vinci Xi platform on bodies donated to science, an integration that remains less documented than dry-lab or virtual-reality curricula (1). Here, the da Vinci platform was not an isolated technological device but part of a high-fidelity educational environment made possible by anatomical infrastructure and body donation, an approach particularly relevant to emerging fields such as robotic cranial neurosurgery, where dedicated training models are considered essential for future clinical translation (31). This model’s replicability, however, depends on the local regulatory and infrastructural conditions that make body donation-based training possible in the first place. Legal frameworks governing the use of donated bodies for surgical training vary substantially across countries, and donor-based simulation programs remain concentrated in settings with well-established willed-body donation systems, while trainees elsewhere may face the added burden of traveling abroad to access equivalent training (32, 33). Italy’s Law 10/2020 provides a specific and comparatively favorable regulatory basis for the Anatomy Center of the University of Bologna institutional program, and this context should be considered when evaluating the generalizability of this training model to healthcare systems without equivalent legal frameworks or donation infrastructure.

Despite this variability, these findings reinforce the broader educational and ethical role of body donation. Body donors are increasingly recognized as “silent teachers” (12), whose contribution can extend beyond traditional dissection to technologically advanced training environments that are realistic, safe, and ethically grounded. This perspective is relevant to a recent debate in the New England Journal of Medicine, which framed anatomical dissection and technological learning tools as competing approaches to anatomy education (34). The present study, together with previous work from the same institutional context (16, 35), challenges this binary framing: body donation and technological innovation can and should be deliberately combined to create a simulation environment that is anatomically realistic, technically advanced, and ethically grounded.

## Conclusion

This pilot study suggests that a robotic surgery training session, combining dry-lab and wet-lab practice on the da Vinci Xi platform within a body donation setting, was perceived as highly valuable by residents and surgeons. Participants reported highly positive evaluations across all course appraisal items, and the qualitative feedback linked this perceived value to hands-on robotic practice, anatomical realism, and exposure to an operative-like workflow. This interpretation was further supported by the finding that participants’ agreement on the educational value of body donation, and their perception of its ethical importance, were both significantly correlated with their overall evaluation of the course. As a pilot evaluation based on self-reported perceptions, this study cannot support conclusions about skill acquisition, competence development, retention, or transferability to clinical practice. Within these limits, the findings support the educational relevance of integrating robotic surgery and body donation. This model offers a setting in which robotic technical skills can be integrated with the anatomical complexity and variability of the human body, team dynamics, and the scientific and ethical value of body donation. More broadly, this experience supports the view that technological innovation and body donation are not competing paradigms in surgical education, but mutually reinforcing ones.

### Limitations

This study has several limitations. It was conducted at a single academic center with an established body donation infrastructure, which may limit generalizability to centers without similar resources. The design did not include a pre/post assessment or a control group, precluding any inference about learning or improvement over time; the absence of a control group reflects the practical constraints of delivering hands-on training to residents and surgeons rather than a correctable design choice. No objective measures of technical performance (e.g., validated Global Rating Scales) or independent evaluators were used; all findings are based on participants’ self-reported perceptions, collected through a study-specific questionnaire that has not been formally validated. A pre/post benchmark assessment using basic simulator-derived metrics was not included in the present study design; incorporating such measures around the wet-lab session would complement self-reported perceptions with objective data and is recommended for future iterations of this training model. This pilot session was not structured as a formal curriculum with defined learning objectives, competencies, assessment strategy, or progression criteria; development of these elements would be a necessary step before this training model could be formally evaluated as part of a structured curricular pathway. The consistently high Likert-scale ratings produced a pronounced ceiling effect, which limits the interpretability of correlations and subgroup comparisons; these analyses should accordingly be regarded as exploratory and hypothesis-generating rather than confirmatory. Self-reported evaluations may also be subject to social desirability bias and a novelty or prestige effect associated with a newly introduced training opportunity, and participation may reflect selection bias toward already motivated trainees. Prior robotic experience was not systematically assessed through the questionnaire, and other potential confounders, including years of training and actual console time, were not formally measured. No follow-up data were collected to assess whether perceived value was sustained over time or associated with subsequent clinical performance. Taken together, these limitations mean that no conclusions can be drawn regarding actual skill acquisition, competence development, retention, or transferability to the operating room; this study should be interpreted as an exploratory pilot evaluation of perceived training value.

## Data Availability

The original contributions presented in the study are included in the article/Supplementary material, further inquiries can be directed to the corresponding author.
