# Supplementary material for: Integrating robotic surgery training and the educational value of body donation: insights from an Italian pilot project on perceived training value
Source: Front Med (Lausanne). 2026 Aug 26;13:1907504. doi: 10.3389/fmed.2026.1907504 (PMC13553216; doi:10.3389/fmed.2026.1907504)
Supplement: Supplementary file 1 [file Table_1.docx]

**Supplementary Material**

Questionnaire

| What is your age? |  |
| --- | --- |
| What is your gender? | 🞏 Male  🞏 Female  🞏 Other  🞏 I prefer not to answer |
| What is your nationality? |  |
| What is your profession? | 🞏 Resident  🞏 Surgeon |
| What is your specialization? | 🞏 General Surgery  🞏 Urology |
| Before today, have you ever taken part in any courses involving bodies donated to science? | 🞏 Yes  🞏 No |
| What were you expecting from this experience in the anatomy lab? |  |
| On a scale from 1 to 5, how would you rate the course? | 2  3  4  5  Very poor Poor Fair Good Excellent |
| On a scale from 1 to 5, how well did the course meet your expectations? | 1  1  2  3  4  5  Not at all Slightly Moderately Very Extremely |
| On a scale from 1 to 5, how useful do you think the course was for your skills? | 1  2  3  4  5  Not at all Slightly Moderately Very Extremely |
| On a scale from 1 to 5, how much would you like to take part in this type of course again? | 1  2  3  4  5  Not at all Slightly Maybe Very Extremely |
| On a scale from 1 to 5, how likely would you be to recommend this type of course to a colleague? | 1  2  3  4  5  Not at all Slightly Maybe Very Extremely |
| On a scale from 1 to 5, how much do you agree with the sentence "Body donation represents a gold standard for medical/education and training"? | 1  2  3  4  5  Strongly Disagree Neither Agree Strongly  disagree agree nor agree  disagree |
| On a scale from 1 to 5, how ethically important do you consider body donation for medical/surgical education and training? | 1  2  3  4  5  Not at all Slightly Moderately Very Extremely |
| Do you have any suggestions or comments? |  |

Questionnaire codebook

| Age | 25-30 = 1  31-35 = 2  36-40 = 3  >40 = 4 |
| --- | --- |
| Gender | Male = 1  Female = 2  Other = 3  I prefer not to answer = -1 |
| What is your nationality? | Italian = 1  Any other nationality = 2 |
| What is your profession? | Resident = 1  Surgeon = 2 |
| What is your specialization? | General Surgery = 1  Urology = 2 |
| Before today, have you ever taken part in any courses involving bodies donated to science? | Yes = 1  No = 2 |

Verbatim responses to open-ended questionnaire items (Q1 and Q9)

*Supplementary Table 1. Verbatim responses to the two open-ended questionnaire items (Q1 and Q9). Dashes indicate no response was provided by that participant.*

| **Participant ID** | **Q1 – What were you expecting from this experience in the anatomy lab?** | **Q9 – Do you have any suggestions or comments?** |
| --- | --- | --- |
| 1 | Improve my basic surgical skills | — |
| 2 | Training and growth | — |
| 3 | Learn and practice | — |
| 4 | Deepen the use of the robot in general surgery and improve practical skills | — |
| 5 | Professional growth | — |
| 6 | Improvement of knowledge | — |
| 7 | Acquisition of robotic surgery experience by trainees | — |
| 8 | Great experience with the robot | — |
| 9 | Learn the surgical technique | — |
| 10 | Focus on anatomy and urologic robotic surgery | — |
| 11 | Teach surgical technique and identify anatomy | — |
| 12 | — | Include a theoretical component on surgical technique in the course |
| 13 | Opportunity to practically apply the use of the robot on human tissue | — |
| 14 | — | — |
| 15 | — | — |
| 16 | — | — |
| 17 | — | More time |
| 18 | — | — |
| 19 | — | — |
| 20 | Professionalism, realistic simulation of surgery | Excellent organization, great opportunity |
| 21 | — | — |
| 22 | — | It would be useful to provide supports or braces to keep the limbs in place |
| 23 | — | Extend the duration of the wet lab |
| 24 | — | More time for cadaver practice |
| 25 | Fantastic | Continue offering this type of activity |
| 26 | Operative practice on cadaver | — |
| 27 | Practical experience in robotics | Increase similar initiatives |
| 28 | — | I would increase the duration of the course |
| 29 | — | — |
| 30 | Learn and practice | It would be fantastic to maintain this course also for senior residents in the coming years |
| 31 | First experience at the robotic console on a human | If possible, offer it more frequently, with a higher number of sessions. Thank you |
| 32 | First experience at the robotic console on a human | Greater number of sessions |
| 33 | Exactly what happened: a highly educational and useful course | — |
| 34 | Gain experience in robotic surgery | Everything excellent |
| 35 | — | — |
| 36 | Simulate robotic surgery on a living patient | More time for practice |
| 37 | A trial exactly like this, as on a living person | Try to provide more time for practice |
| 38 | — | Offer more courses |
| 39 | Have a surgical experience as close to reality as possible | Organize similar events more often |
| 40 | Improve surgical technique | — |
| 41 | — | — |

SPSS outputs

*Supplementary Table 2. Sample descriptive statistics*

*Supplementary Table 3. Sample gender frequency*

*Supplementary Table 4. Sample nationality frequency*

*Supplementary Table 5. Sample profession frequency*

*Supplementary Table 6. Sample specialization frequency*

*Supplementary Table 7. Sample frequency for Q1*

*Supplementary Table 8. Likert items descriptive statistics*

*Supplementary Table 9. Sample frequency for Q2*

*Supplementary Table 10. Sample frequency for Q3*

*Supplementary Table 11. Sample frequency for Q4*

*Supplementary Table 12. Sample frequency for Q5*

*Supplementary Table 13. Sample frequency for Q6*

*Supplementary Table 14. Sample frequency for Q7*

*Supplementary Table 15. Sample frequency for Q8*

*Supplementary Table 16. Internal consistency for Q2-Q6*

*Supplementary Table 17. Likert items descriptive statistics*

*Supplementary Table 18. Training value index descriptive statistics*

*Supplementary Table 19. Sample frequency for training value index*

*Supplementary Table 20. Spearman’s correlation matrix*

*Supplementary Table 21. Results of Mann-Whitney test between training value index and gender*

*Supplementary Table 22. Results of Mann-Whitney test between training value index and nationality*

*Supplementary Table 23. Results of Mann-Whitney test between training value index and profession*

*Supplementary Table 24. Results of Mann-Whitney test between training value index and specialization*

*Supplementary Table 25. Results of Mann-Whitney test between training value index and previous experience*

*Supplementary Table 26. Results of Mann-Whitney test between Q7/Q8 and gender*

*Supplementary Table 27. Results of Mann-Whitney test between Q7/Q8 and nationality*

*Supplementary Table 28. Results of Mann-Whitney test between Q7/Q8 and profession*

*Supplementary Table 29. Results of Mann-Whitney test between Q7/Q8 and specialization*

*Supplementary Table 30. Results of Mann-Whitney test between Q7/Q8 and previous experience*
